# Impact of Opera on Mental Health Stigma: Pilot of Provider/Community Workshop

**DOI:** 10.1007/s10597-021-00908-4

**Published:** 2021-12-01

**Authors:** Kia Skrine Jeffers, Joseph D. Mango, Lingqi Tang, Elyn R. Saks, Kenneth B. Wells, Bowen Chung

**Affiliations:** 1 UCLA School of Nursing, 700 Tiverton Avenue, Los Angeles, CA 90095 USA; 2 Center for the Study of Racism, Social Justice & Health, UCLA Fielding School of Public Health, Los Angeles, CA USA; 3 HEArts Program, Center for Health Services and Society, UCLA Jane and Terry Semel Institute for Neuroscience and Human Behavior, Los Angeles, CA USA; 4 Center for Health Services and Society, UCLA Jane and Terry Semel Institute for Neuroscience and Human Behavior, Los Angeles, CA USA; 5 Psychology, and Psychiatry and the Behavioral Sciences, USC Gould School of Law, Los Angeles, CA USA; 6 Saks Institute for Mental Health Law, Policy and Ethics, USC Gould School of Law, Los Angeles, CA USA; 7 Psychiatry and Biobehavioral Sciences, UCLA David Geffen School of Medicine, Los Angeles, CA USA; 8 Health Policy and Management, UCLA Fielding School of Public Health, Los Angeles, CA USA; 9 Greater Los Angeles Veterans Administration Health System, Los Angeles, CA USA; 10 Los Angeles County Department of Mental Health, Los Angeles, CA USA; 11 Department of Psychiatry, Harbor-UCLA Medical Center, Los Angeles, CA USA

**Keywords:** Opera, Mental health stigma, Pilot evaluation

## Abstract

**Background:**

Arts can influence mental health stigma, but little is known about impact of operas. We examined effects of a two-opera workshop on complicated grief and schizophrenia.

**Methods:**

Pre-post audience surveys with post-workshop discussion. The primary outcome was a 4-item measure of willingness to engage with persons with grief or schizophrenia. Secondary outcomes were perceptions of art affecting stigma and stigma mediators. Of 47 participants, 33 had pre-post surveys for both operas.

**Results:**

There was a significant pre-post opera increase in audience willingness to engage with persons with grief or schizophrenia (*p* < .001). Perceptions of impact on mediators such as empathy, were significantly greater for the opera on schizophrenia relative to grief (*p* < .001)..

**Conclusion:**

The pre- to post increase in audience willingness to engage with affected persons (primary) with greater impact on secondary mediators for the schizophrenia opera and post-discussion suggest that operas may be a forum for addressing mental health stigma and promoting empathy.

## Introduction

### Background

There is substantial literature on use of arts on mental health themes for therapy and public and provider education, with some research on impacts on mental health stigma and other outcomes, largely through mixed methods (Estroff et al., 2004; Fancourt & Finn, 2020; Heenan, 2006; Lenette et al., 2016; McLean et al., 2011; Ørjasæter et al., 2017; Torrissen, 2015; Warren, 2016). There are similarities and differences in use of arts for creative and therapeutic purposes, such as arts permitting more psychological distance from the subject matter relative to therapy (Ayers et al., 2003; Margrove et al., 2013). Addressing social stigma of mental health for providers, patients and the general public may be important, as stigma is associated with less engagement in treatment and worse outcomes for those needing services (Henderson et al., 2013; Parcesepe & Cabassa, 2013). There are quantitative and qualitative studies providing evidence of positive effects of arts events on stakeholders’ health, reducing stigma or discrimination and improving attitudes toward mental health/use of mental health services (Fancourt & Finn, 2020; Gronholm et al., 2017; Hacking et al., 2006; McLean et al., 2011; Parcesepe & Cabassa, 2013). Effect sizes were modest and of short duration, but qualitative studies suggest potential for longer impact (Michalak et al., 2014).

There are few studies of impacts of operas on mental health themes (Fancourt & Finn, 2020). Opera is regarded as a complex art form, combining music, poetry, and drama. Our goal was to develop a pilot evaluation of an opera workshop including excerpts from two mental health-themed operas and post-audience discussion, on stigma-related outcomes.

This project was developed as part of the Healing and Education through the Arts (HEArts) program (Mango et al., 2018), designed to promote stakeholder participation in developing and evaluating art projects that address mental health, to address mental health stigma and promote help-seeking. Two opera projects, “The First Lady” and “The Center Cannot Hold, Part I: The Illness” focused on complicated grief and schizophrenia, respectively. “The First Lady” was based on the life of Eleanor Roosevelt following the death of her husband, President Franklin Roosevelt (FDR); and “The Center Cannot Hold Part I: The Illness” focused on recovery from serious mental illness based on the memoir of Elyn Saks, (Saks, 2007) University of Southern California (USC) law professor. Each opera was produced at UCLA, with the latter streamed online after the workshop (Groves, 2010; Willich, 2017).

### Framework

**Fig. 1:**
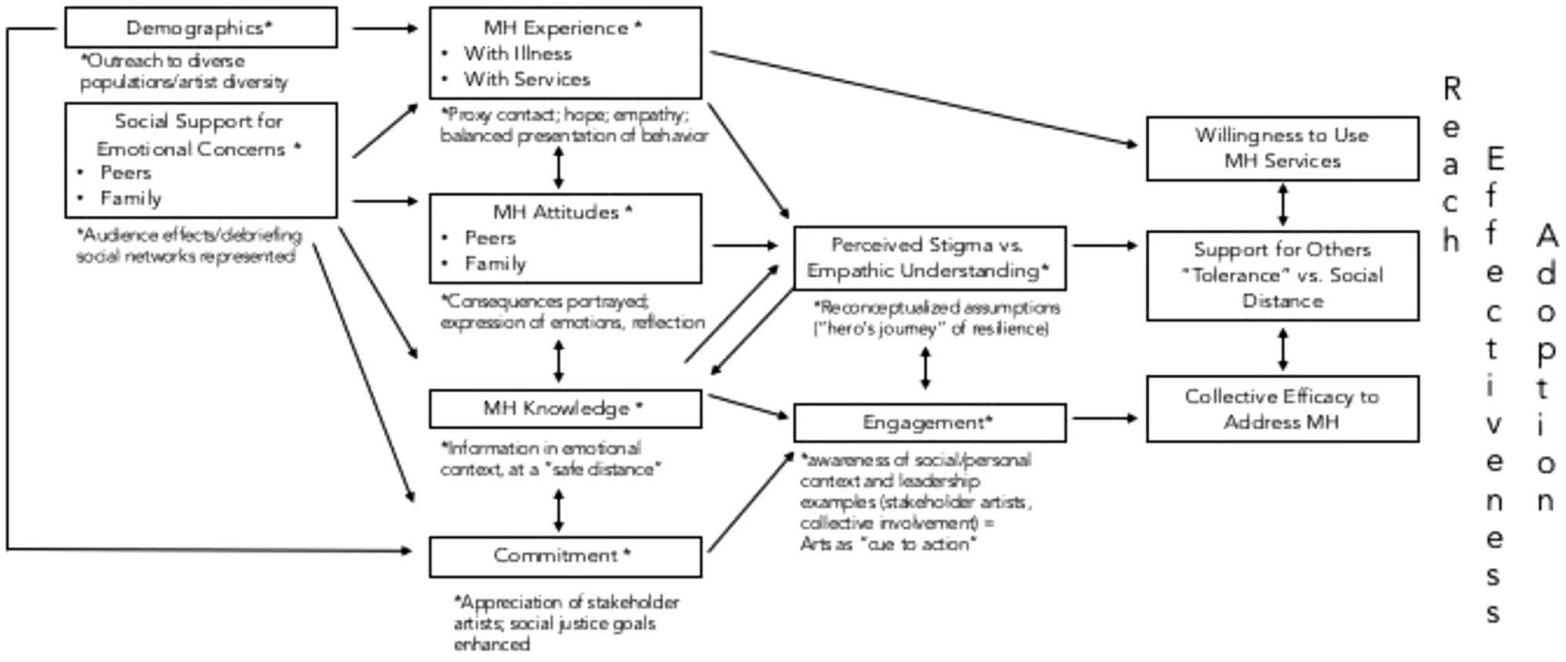
Framework for arts events affecting stigma and social action (Mango et al., 2018)

The HEArts program is guided by a conceptual framework that shows the relationships among theatrical arts, mental illness stigma, and help-seeking (Fig. 1) (Mango et al., 2018). This framework informed development of the operas and pilot evaluation. Specifically, by art events illustrating key issues (e.g., grief and schizophrenia), demonstrating value of treatment and social support, and increasing knowledge and empathy, stigma related to mental illness may be reduced. Narrative characteristics such as a “heroine’s journey” may enhance effects (Allport, 1954; Patterson & Sextou, 2017; Pinfold et al., 2005; Quinn et al., 2011).

### Goals

Within this framework, we sought to evaluate the impact of a two-opera workshop using excerpts from “The First Lady” and “The Center Cannot Hold,” including audience discussions with an author/stakeholder/protagonist (Elyn Saks), the opera’s creators, performing artists, local providers and community partners. Thus, the evaluation pertains to the workshop as a whole, including music excerpts and discussion.

### Hypotheses

The primary outcome of this pilot evaluation was a measure of audience members’ intentions to interact with individuals with grief and/or schizophrenia as a measure of stigma reduction. Secondary outcomes were perceptions of art to reduce stigma, and proposed mediators of effects of art on stigma, based on measures from a play evaluation (Mango et al., 2019). We hypothesized that the workshop would increase audience members’ willingness to engage with persons experiencing grief or schizophrenia. Our exploratory hypothesis was that the workshop would enhance audience perceptions of effects of arts events on mental health stigma. Given that schizophrenia is less common (less than 2% of the population) than grief (affecting many in the population), (Kessler et al., 2005; Lundorff et al., 2017; Wu et al., 2006) and that the event included live discussion with the schizophrenia story protagonist/author, an exploratory hypothesis was of stronger audience response for mediators of art event effects on stigma for “The Center Cannot Hold” (schizophrenia) compared to “The First Lady” (grief).

## Methods

### Design

This pilot study utilized written, pre- and post- surveys of the workshop overall and for two operas. We included a post-only, open-ended comment option. The surveys were distributed with a written explanation and informed consent read by faculty not involved with the production. Participation was voluntary and anonymous, with data collection approved by UCLA’s Institutional Review Board.

### Workshop Format

The workshop was advertised through UCLA and community partners (Healthy African American Families II and Los Angeles County Department of Mental Health Services). The workshop was free and co-hosted by a local foundation and HEArts at the UCLA Jane and Terry Semel Institute for Neuroscience and Human Behavior on the UCLA Health campus. The psychiatrist Institute director introduced the workshop; the composer provided a framework for the operas; and excerpts of both operas were performed by soloists with piano accompaniment. Excerpts of “The First Lady” illustrated Eleanor Roosevelt’s concern with FDR’s betrayal prior to his death (i.e., with his mistress, Eleanor’s former secretary), responses of Eleanor, her daughter (who supported that relationship), FDR’s mistress, and FDR’s attendant. The narrative conveyed Eleanor’s journey from learning of FDR’s death to forgiveness of her daughter (for promoting the affair), with an understanding of the value of love for all main characters. Excerpts from “The Center Cannot Hold” illustrated Elyn Saks’ experience with psychotic symptoms; responses to involuntary hospitalization as a Yale law student; experiences of her providers; Elyn’s movement toward recovery and developing a key friendship; and graduating from law school, while coping with being separated from her friend. The workshop concluded with a premiere of the opera’s sequel, as Elyn joined the USC faculty and met the librarian she later married. After excerpts, an award was presented to the composer, with a talk by Elyn Saks on recovery from schizophrenia, audience discussion and completion of the post-event survey.

### Measures

The pre-event survey included information on demographics (age, gender, race/ethnicity, education), being a mental health or other provider (dichotomized as provider or not), and 4 items on audience members’ personal experience with grief and schizophrenia—i.e., with another person, or as provider or caretaker (each yes/no).

#### Primary Outcome

There were 4 items on willingness to engage socially or as a provider/support for persons with complicated grief, or with schizophrenia (responses 1 to 5, strongly disagree to strongly agree) with mean score analyzed.

#### Secondary Outcome

There were 2 items on the effectiveness of opera to increase understanding of mental illness and art in general to address stigma of mental illness (responses 1 to 5, strongly disagree to strongly agree) analyzed by mean score.

#### Exploratory Outcomes

Post-event surveys included 8 items on how well each opera portrayed factors that mediate effects on stigma (importance of social/family support, experience of illness, importance of hope, “heroine’s journey” to resilience/recovery; understanding and empathy; commitment to helping others; importance of seeking help or support; personal consequences of social stigma); and 5 items on how the overall event affected people (i.e., more sympathetic towards people with complicated grief or severe mental illness; more comfortable around such persons; feeling concern about these issues in themselves or others; feeling comfortable talking about grief or severe mental illness; reaching out to offer support to someone in distress, or “humanistic” factors) (responses 1 to 5, strongly agree), each set analyzed by mean score.

#### Open-Ended

The post-survey included an item: “If you would like to share more about how the opera scenes affected you, please share below.”

### Sample

47 individuals completed any item, 44 completed any post-event item with 40 for “The First Lady,” 41 for “The Center Cannot Hold,” and 33 for both operas; with 12 legible open-ended responses.

### Analysis

We describe the distribution of pre-survey responses, and present distributions stratified by provider status using Chi-square tests for categorical data and Wilcoxon Mann-Whiney tests for mean differences across provider status. For primary and secondary outcomes noted above, we provide unadjusted tests of pre-post change scores using Wilcoxon 1 sample signed-rank test but discuss multiple comparisons. We provide standardized effect sizes for comparisons, calculating mean difference divided by standard deviation of mean difference. We considered mean change in intentions to engage with individuals with grief/schizophrenia as the primary outcome; and as secondary mean change in perception of opera/arts to increase understanding/address stigma of mental illness. We compared change in mean pre and post scores/opera for participants responding to both surveys; and for sensitivity analysis, limited to those completing responses on both operas. We compared responses for how well each opera portrayed stigma-related mediator effects (mean of 8 items per opera), the difference between operas as an exploratory analysis, and describe impact on mean of 5 “humanistic” items and individual items to inform future research. For open-ended responses, we grouped by type of response (negative, positive, mixed), identified main themes and illustrative quotes to complement main survey findings, following a rapid analysis method (Gale et al., 2019).

## Results

### Audience Characteristics

Mean age among audience members was 51 years, and 62% identified as female (Table 1). Higher education levels were observed for healthcare providers compared to non-providers, with all having high school diploma/GED or higher. There was racial/ethnic diversity, with 17% self-identifying as Hispanic/Latino, 10.6% African American, 44.7% non-Hispanic white, and 27.7% other race/ethnicity. The majority experienced or knew someone with grief, and providers were almost twice as likely to have provided services for persons with complicated grief compared to non-providers (*p* = 0.017). Over half had or knew people with severe mental illness. Overall, participants agreed (mean = 4.6, SD = 0.6) that viewing opera/arts increases understanding or reduces mental health stigma. Willingness to engage or provide services was moderately high (mean = 3.5, SD = 0.6), with no significant difference between providers and non-providers (*p* > 0.10).

**Table 1:** Pre-workshop Audience Background and Attitudes

	*n*	Overall (*n* = 47)	Healthcare provider (*n* = 29)	Not a healthcare provider (*n* = 18)	*p* value^†^
Age, mean (SD), years	45	52.3 ± 16.7	51.9 ± 14.2	50.1 ± 18.2	.833
Female (versus male, none reported other)	47	61.7%	65.2%	64.7%	.973
Education (none less than high school)	40				.002
High School (none reported less than high school)		5.0%	0.0%	11.8%	
Some College		10.0%	0.0%	23.5%	
Bachelor Degree		17.5%	8.7%	29.4%	
Master/Doctoral		67.5%	91.3%	35.3%	
Race/Ethnicity (hierarchy order)	47				.823
Any Hispanic		17.0%	21.7%	17.6%	
Any Black or African American (not Hispanic)		10.6%	8.7%	17.6%	
Any White/Caucasian (not Hispanic or African American)		44.7%	43.5%	35.3%	
Other (Not Hispanic, African American or White)		27.7%	26.1%	29.4%	
Have you or someone you know ever experienced complicated grief? (yes/no)	47	85.1%	82.6%	88.2%	.622
Have you supported or provided services for someone with complicated grief? (yes/no)	47	61.7%	78.3%	41.2%	.017
Have you or someone you know ever had schizophrenia or severe mental illness (yes/no)	45	64.4%	65.2%	56.3%	.571
Have you have supported or provided services for someone with schizophrenia (yes/no)	45	53.3%	60.9%	37.5%	.151
Number of yes responses to grief/support, range 0–4	45	2.6 ± 1.2	2.9 ± 1.1	2.3 ± 1.2	.097
Attitudes (1–5 strongly disagree to strongly agree)					
Mean agreement score, opera/arts increase understanding/reduce stigma (2 items)	45	4.6 ± 0.6	4.5 ± 0.6	4.5 ± 0.6	.973
Mean willingness to engage score (grief, schizophrenia) 4 items	46	3.5 ± 0.6	3.5 ± 0.5	3.5 ± 0.6	.872

Data are presented as mean ± standard deviation (SD) for continuous variables, % for categorical variables, variation in *n* across variables results from missing data

^†^For the difference between healthcare provider and not healthcare provider groups, based on Wilcoxon-Mann–Whitney test for continuous variables and Chi-square test for categorical variables

### Pre–post Impact

There was a statistically significant pre-post increase in willingness to engage socially or as a provider with persons with complicated grief and/or schizophrenia (primary) (*p* < 0.001, standardized effect size (SE) 0.6), including for persons with data for both operas (*p* = 0.006, SE 0.51) (Table 2)—significant considering multiple comparisons. For the secondary outcome “opera/arts increase understanding/reduce stigma,” there was a borderline trend toward agreement (whole sample, *p* = 0.064; with data on both operas, *p* = 0.07). The individual item with a significant change was “Arts can reduce social stigma of mental illness” (*p* = 0.012), an exploratory finding significant with up to 4 multiple comparisons.

**Table 2:** Pre-post audience change in attitudes

	*n*	Pre Mean (95%CI)	Post Mean (95% CI)	Difference Mean (95% CI)	*p*^†^	ES^‡^
Primary (How willing would you be to, 1–5, 5 +)						
Make friends or socialize with someone suffering from complicated grief	44	3.6 (3.4, 3.8)	3.7 (3.6, 3.9)	0.1 (0, 0.3)	.07	0.33
Make friends or socialize with someone suffering from schizophrenia	43	3.3 (3.1, 3.6)	3.6 (3.4, 3.8)	0.3 (0.1, 0.4)	< .001	0.61
Support or provide services to someone with complicated grief	44	3.6 (3.5, 3.8)	3.7 (3.6, 3.9)	0.1 (-0.1, 0.2)	.398	0.17
Support or provide services to someone with schizophrenia	43	3.6 (3.3, 3.8)	3.7 (3.6, 3.9)	0.2 (0, 0.3)	.016	0.44
Mean willingness to engage score (grief, schizophrenia) 4 items	43	3.5 (3.4, 3.7)	3.7 (3.6, 3.9)	0.2 (0.1, 0.3)	< .001	0.60
Mean willingness to engage score (grief, schizophrenia) 4 items; among those rating both operas pre/post	33	3.6 (3.4, 3.8)	3.7 (3.5, 3.9)	0.1 (0, 0.2)	.006	0.51
Secondary (In your opinion, agreement, 1–5, 5 +)						
Watching an opera or musical can increase understanding of emotional stress or mental illness and increase empathy	43	4.6 (4.4, 4.8)	4.7 (4.6, 4.9)	0.1 (-0.1, 0.3)	.364	0.18
The arts can reduce social stigma of mental illness	41	4.6 (4.4, 4.8)	4.8 (4.7, 4.9)	0.2 (0.1, 0.4)	.012	0.46
Mean agreement score, opera/arts increase understanding/reduce stigma, 2 items	41	4.6 (4.4, 4.8)	4.8 (4.6, 4.9)	0.2 (0, 0.3)	.064	0.33
Mean agreement opera/arts increase understanding/reduce stigma, 2 items among those rating both operas pre/post	31	4.6 (4.4, 4.8)	4.8 (4.6, 4.9)	0.2 (0, 0.4)	.07	0.37

^†^Wilcoxon Signed-Rank test

^‡^standardized effect size

### Post-Only Outcomes

For mediating features of arts affecting mental health stigma, mean score for “The First Lady” was 4.2 (95% CI, 3.9–4.5) and 4.6 (4.3–4.9) for “The Center Cannot Hold” among those rating both (Table 3), with a significantly higher mean score for “The Center Cannot Hold” (difference of 0.4 points, 95% confidence interval 0.1–0.6; *p* < 0.001; standardized effect size of 0.56), significant considering multiple comparisons. For an exploratory analysis of individual items, there were statistically significant differences (*p* < 0.05) favoring “The Center Cannot Hold” for 5: conveying a “heroine’s” journey to resilience/recovery, increased understanding/empathy, increased commitment to help others, importance of seeking help/support, and conveying personal consequences of social stigma. The mean score was high for the 5 post-only items on overall events’ impact (humanistic values) with mean = 4.5, SD = 0.7, with no difference between providers and non-providers (*p* > 0.10). (Table 4).

**Table 3:** Post rating, audience perception by Opera (The Center Cannot Hold—The First Lady)

Variables	*n*	The First Lady Mean (95%CI)	The Center Cannot Hold Mean (95% CI)	Difference Mean (95% CI)	*p*^†^	ES^‡^
How well did the opera convey (1–5, 5+)						
Importance of social/family support	33	4.4 (4.1, 4.7)	4.6 (4.3, 4.9)	0.2 (-0.1, 0.5)	.131	0.29
Experience of grief/mental illness	33	4.4 (4.1, 4.7)	4.5 (4.2, 4.9)	0.2 (-0.2, 0.5)	.274	0.14
Importance of hope	33	4.3 (4.0, 4.7)	4.6 (4.2, 5.0)	0.3 (-0.1, 0.6)	.140	0.30
A “heroine’s” journey to resilience/recovery	33	4.2 (3.9, 4.6)	4.6 (4.3, 5.0)	0.4 (0.2, 0.7)	.004	0.56
Increase understanding/empathy	33	4.2 (3.8, 4.6)	4.6 (4.2, 4.9)	0.4 (0.1, 0.7)	.023	0.44
Increase commitment to help others	33	4.1 (3.7, 4.4)	4.5 (4.2, 4.8)	0.5 (0.2, 0.7)	.006	0.55
Importance of seeking help or support	33	4.2 (3.8, 4.6)	4.7 (4.4, 5)	0.5 (0.2, 0.8)	< .001	0.65
Personal consequences of social stigma	33	4.1 (3.6, 4.5)	4.6 (4.3, 4.9)	0.5 (0.2, 0.8)	.005	0.55
Mean rating score on 8 items	33	4.2 (3.9, 4.5)	4.6 (4.3, 4.9)	0.4 (0.1, 0.6)	< .001	0.56

^†^Wilcoxon Signed-Rank test

^‡^standardized effect size

**Table 4:** Post-only, audience perception of workshop impact

Has the play moved you in (agreement 1–5, 5+)	*n*	Overall	Not healthcare provider (*n* = 17)	Healthcare provider (*n* = 23)	*p*^†^
Being more sympathetic towards persons with complicated grief or severe mental illness	44	4.5 ± 0.8	4.5 ± 0.8	4.6 ± 0.7	.780
Feeling more comfortable around persons with complicated grief or severe mental illness	44	4.5 ± 0.7	4.4 ± 0.7	4.6 ± 0.7	.403
Feeling less alone with concerns about grief or severe mental illness in yourself or someone you know	44	4.3 ± 0.8	4.2 ± 0.8	4.4 ± 0.7	.527
Feeling more comfortable talking about grief or severe mental illness with someone you know	45	4.3 ± 0.8	4.3 ± 0.7	4.5 ± 0.7	.311
Reaching out more to someone you know to offer support when they are distressed	45	4.5 ± 0.7	4.6 ± 0.6	4.5 ± 0.7	.674
Mean agreement on impact score (mean, 5 items)	42	4.5 ± 0.7	4.4 ± 0.6	4.5 ± 0.6	.613

Data are presented as mean ± standard deviation (SD) for continuous variables

^†^For the difference between healthcare provider and not healthcare provider groups, based on Wilcoxon-Mann–Whitney test

### Open-Ended Response

For 12 legible comments, one was negative about auditory experience (“loud, high pitches, interfering with concentration”), and 11 positive, describing the operas as “moving,” “incredible intersection of illness, art and humanism,” and “a healing experience.” A comment regarding “The Center Cannot Hold” was, “This was a story I knew nothing about – the opera brought to life the story and allowed to understand and enlighten me re: mental illness.” One participant commented on the emotional experience of a family member with limited English proficiency who appeared moved. Another member suggested that there was a “need to show to schools and training.” One commented the discussion with Elyn Saks, “even more humanized her”.

## Discussion

We presented results from a pilot study of impact of a workshop event featuring excerpts from two operas on complicated grief (Eleanor Roosevelt) and serious mental illness, hospitalization and recovery (Elyn Saks), with post discussion with Saks and others. To our knowledge, it is one of the first evaluations of the impact on mental health-related opera events, though there are studies of plays, musical theater and other media (Estroff et al., 2004; Fancourt & Finn, 2020; Heenan, 2006; Lenette et al., 2016; Mango et al., 2019; McLean et al., 2011; Ørjasæter et al., 2017; Torrissen, 2015; Warren, 2016). The workshop was held within a medical center with a diverse audience, more than half non-white, including health care providers and community members. Pre-survey results indicated that most audience members had prior experience with mental health issues and most held high views of the value of art to understand and address mental health stigma. Yet, we found that the workshop was associated with a pre- to post-event increase in the primary outcome of “willingness to engage with people with complicated grief and/or severe mental illness,” with moderate effect sizes and significant at a level considering multiple comparisons with main secondary outcomes. This is promising for future research on arts to address stigma, given impact of stigma on limiting treatment access and outcomes (Henderson et al., 2013; Parcesepe & Cabassa, 2013). The borderline significant increase on perception of opera and art to address stigma would not be significant adjusting for multiple outcomes, which may be partly due to high pre-event values. This increases the importance of the post-only measures on mediators for effects of art on stigma, for which there was a significantly greater average score for “The Center Cannot Hold” on schizophrenia than for “The First Lady” on complicated grief—a finding significant even considering multiple comparisons for main and secondary outcoms. This finding could be due to audiences having less background in schizophrenia than grief, or including the author (Elyn Saks) in post-event discussion, also noted in one open-ended response, which may reinforce the importance of including stakeholders in opera event discussions. For exploratory purposes, we found that individual items significant ranged from more effectively showing a “heroine’s journey”, an increase in empathy, commitment to help others, help or support persons and address personal consequences of social stigma. Several of these factors may affect how arts influence mental health stigma (Allport, 1954; Chandra & Minkovitz, 2007; Mango et al., 2018; Patterson & Sextou, 2017; Pinfold et al., 2005; Quinn et al., 2011).

The open-ended responses, while only 12, included one negative on musical sound, and others largely positive on the “humanism” of the experience, reinforcing that information on schizophrenia was helpful and post-discussion with Elyn Saks effective, consistent with explanations for difference in impact of the operas. Participatory or partnered events sharing lived experience is a featured approach for HEArts (Mango et al., 2018) and may be an important issue for future research on arts in mental health, including opera.

### Limitations

The audience was small, over half were providers in a healthcare auditorium, responses could differ in other settings such as those with less pre-event experience with mental health issues. Further, the survey did include audience health outcomes, and were self-reported. The composer was a faculty member at the institution, which could bias findings toward positive. The presence of the protagonist (and co-librettist) of one opera may alone have had an effect reinforced by one open-ended comment suggesting the importance of stakeholder participation. Other participatory art approaches are known to impact stigma, such as “Playback Theater” (Yotis et al., 2017), “Forum Theater” (Wilson, 2013) and knowledge translation in bipolar disorder (Michalak et al., 2014). We note however that “The First Lady” impact was also positive. While we had one primary outcome, we had secondary and exploratory outcomes, but main comparisons were significant considering multiple comparisons. It will be important for subsequent studies to have larger audiences and examine impacts of stakeholder discussions.

### Conclusion

Overall, this pilot study suggests that it is feasible to present and evaluate operatic materials on mental health ranging from grief to serious mental illness, and evaluate impact with an audience of healthcare providers and community stakeholders. Even with high baseline values of intentions to engage with affected individuals, the workshop was associated with significant mean increases in engagement intentions. This may suggest it is important for provider and public audiences to engage in arts events including opera, for provider and public entertainment and education, and systematically evaluate impacts of such events.
